# Protective Effect of Fucoidan against MPP^+^-Induced SH-SY5Y Cells Apoptosis by Affecting the PI3K/Akt Pathway

**DOI:** 10.3390/md18060333

**Published:** 2020-06-25

**Authors:** Huaide Liu, Jing Wang, Quanbin Zhang, Lihua Geng, Yue Yang, Ning Wu

**Affiliations:** 1 School of Life Sciences, Nantong University, Seyuan Road 9, Nantong 226019, China; hold126@126.com; 2 Key Laboratory of Experimental Marine Biology, Center for Ocean Mega-Science, Institute of Oceanology, Chinese Academy of Sciences, Qingdao 266071, China; qbzhang@qdio.ac.cn (Q.Z.); lhgeng@qdio.ac.cn (L.G.); yueyang@qdio.ac.cn (Y.Y.); wuning@qdio.ac.cn (N.W.); 3 Laboratory for Marine Biology and Biotechnology, Qingdao National Laboratory for Marine Science and Technology, Wenhai Road, Aoshanwei, Jimo, Qingdao 266237, China

**Keywords:** fucoidan, sulfated heterosaccharide, dopamine neurons apoptosis, PI3K–Akt

## Abstract

The main pathologic changes of the Parkinson’s disease (PD) is dopaminergic (DA) neurons lost. Apoptosis was one of the important reasons involved in the DA lost. Our previous study found a fucoidan fraction sulfated heterosaccharide (UF) had neuroprotective activity. The aim of this study was to clarify the mechanism of UF on DA neurons using human dopaminergic neuroblastoma (SH-SY5Y) cells a typical as a PD cellular model. Results showed that UF prevented MPP^+^-induced SH-SY5Y cells apoptosis and cell death. Additionally, UF pretreated cells increased phosphorylation of Akt, PI3K and NGF, which means UF-treated active PI3K–Akt pathway. Moreover, UF treated cells decreased the expression of apoptosis-associated protein, such as the ratio of Bax/Bcl-2, GSK3β, caspase-3 and p53 nuclear induced by MPP^+^. This effect was partially blocked by PI3K inhibitor LY294002. Our data suggested that protective effect of UF against MPP^+^-induced SH-SY5Y cells death by affecting the PI3K–Akt pathway. These findings contribute to a better understanding of the critical roles of UF in treating PD and may elucidate the molecular mechanisms of UF effects in PD.

## 1. Introduction

Parkinson’s disease (PD) is a neurodegenerative disease characterized by the selective loss of dopaminergic (DA) neurons and the formation of Lewy bodies in the brain. The mainly symptoms was movement disorders such as bradykinesia, myotonia, tremors and abnormal gait [1]. Many reasons such as apoptosis, oxidative stress, genetic factors, environmental factors, mitochondrial dysfunction, ubiquitin-proteasome system dysfunction, immune abnormalities, excitotoxicity and cytotoxicity of calcium may be related to the occurrence of PD [2,3]. Many studies have shown that DA neuron apoptosis has an important effect on the pathogenesis of PD, the number of apoptotic cells in PD patients is nearly 10 times more than normal aged person [4]. However, the exact cause of DA neuron apoptosis is unknown [5]. Oxidative stress, loss of antioxidant function and mitochondrial function damage, can induce DA neuron apoptosis at different levels [6]. The inhibition of pro-apoptotic protein Bax and anti-apoptotic protein Bcl-2 can regulate the apoptosis of DA neurons [7]. Therefore, regulation the expression of apoptosis-associated genes and proteins has become another strategy for the treatment of PD.

The PI3K–Akt pathway plays an important role in neuronal survival and death. Activation the PI3K–Akt pathway can inhibit the activity of downstream caspase-3 and thus inhibit the apoptosis of DA neurons, which can be weakened by the PI3K-specific inhibitor LY294002 [8]. Studies found pretreatment with simvastatin, sulforaphane, erythropoietin, β-interferon and catechins on 6-OHDA-damaged SH-SY5Y cells can increase the PI3K phosphorylation and directly activates the PI3K signaling pathway. Activated Akt can inhibit the activity of downstream caspase-3 and thus inhibit the apoptosis of DA neurons, which can be weakened by the PI3K-specific inhibitor LY294002 [9]. The brain tissues of PD patients and normal people were analyzed by immunofluorescence and western blotting after death. It was found that Akt and activated phosphoSer473-Akt were significantly reduced in the brains of PD patients. Nerve growth factor (NGF) plays an important role in the stages of neuron growth and development, axon growth, transmitter synthesis and cell apoptosis. Studies have shown that the application of corresponding treatments after spinal cord ischemia can induce NGF to activate the PI3K–Akt pathway to inhibit neuronal apoptosis. It can be seen that increase NGF to activate the PI3K–Akt pathway to inhibit DA neuronal apoptosis is a new strategy for the prevention and treatment of PD.

Marine seaweeds produce and accumulate a large number of substances with special chemical structures, physiological activities and functions during their growth and metabolism. The development and utilization of marine biologic resources is an important area and direction for drug candidates. The main feature of the polysaccharides extracted from seaweeds is that it contained sulfated groups. The structures were more complex and the activities were more excellent compared with the polysaccharides extracted from the land plants [10]. Fucoidan is a kind of sulfated polysaccharide extracted from brown algae. It has various biologic activities such as antivirus, antitumor, antimutation, antiradiation and immunity enhancement [11]. The bioactivity such as antioxidant, anticoagulation, neuro-protective activity of fucoidan depends on several structural parameters such as the degree of sulfation (DS), the molecular weight, other substitution groups and position, type of sugar and glycosidic branching. Our preliminary study had shown that the fucoidan with highest sulfated group exhibited stronger activity in scavenging superoxide radical and also hydroxyl radical [12]. The sulfated and benzoylated derivatives of fucoidan could enhance the neuroprotective activity by increasing mitochondrial activity and decreasing LDH and ROS release induced by 6-OHDA (*p* < 0.01 or *p* < 0.001) [13]. Our previous studies found that fucoidan (FPS) can reduce DA neurons damage in phenyl-1,2,3,6-tetrahydropyridine (MPTP)-induced PD mouse model [14,15]. FPS has a protective effects on oxidative damage and inflammatory lesion on DA neurons caused by MPTP in PD mouse, [16]. FPS is a crude polysaccharide prepared from *Saccharina japonica*. After degradation and purification, we got three fractions with different sulfated groups and monosaccharides compositions. Among the samples, UF with highest uronic acid and lowest sulfated groups has the strongest neuroprotective effect both in vitro and in vivo [17]. UF can increase the level of antioxidant enzymes and reduce the level of lipid peroxidation in PD mice [18]. We further found that UF can upregulate the expression of the anti-apoptotic protein Bcl-2, reduce the expression of the pro-apoptotic protein Bax, and significantly inhibit the apoptosis of DA neurons in H_2_O_2_–induced SH-SY5Y cell model [13]. Therefore, we speculate that UF has effect on the DA neurons apoptosis. However, it is not yet established whether UF exhibited apoptosis activity through PI3K–Akt pathway in a MPP^+^ induced neuronal cell line. Can UF activate PI3K by acting on the NGF to cause progressive activation of the PI3K–Akt pathway? How does UF regulate downstream signaling molecules and proteins in the PI3K–Akt pathway?

The aim of this study is to clarify whether UF can activate the PI3K–Akt pathway by acting on NGF protein and illuminate the anti-apoptosis mechanism of UF through the PI3K–Akt pathway. This study will provide experimental foundation and theoretical basis for the application of UF in PD therapy and provide a scientific basis for the development of new and effective anti-PD marine drugs.

## 2. Result

### 2.1. Chemical Analysis

The chemical composition of FPS, DF (low molecular weight fucoidan) and UF are shown in Table 1. All these three samples were sulfated heteropolysaccharides; they were mainly made of fucose, uronic acid and sulfated groups. However, the exactly chemical composition were different. The chemical composition of FPS and DF were nearly the same, however. The molecular weight of FPS was 10 times higher than DF. Among the three samples, UF had the highest uronic acid and lowest sulfated groups. The monosaccharides of three samples all contained fucose, galactose, glucose, rhamnose, mannose and xylose. Fucose was the mainly monosaccharide in all three samples, followed with galactose The structure of FPS, DF and UF were published by our group. The backbone of FPS and DF was similar: they were primarily made by (1→3)-linked α-l-fucopyranose residues and a few (1→4)-α-L-fucopyranose linkages. β-d-galactose and fucose were linked at the branch points at C-4 or C-2 of α-L-fucopyranose residues. Sulfate groups occupied at C-4 or C-2, sometimes C-2, 4 to fucose residues and C-3 and/or C-4 to galactose residues. The structure of UF was more complex. The backbone was made by 4-linked uronic acid and 2-linked mannose. The branch chain was composed of 1–3-linked fucose, 1–6-linked galactose and 1–4-linked glucuronic acid. The sulfate group was connected to the C-6 position of mannose. and C-4 or C-2 position of fucose [19].

### 2.2. Protective Effect of UF on MPP^+^-Induced Neurotoxicity in SH-SY5Y Cells

**Figure 1:**
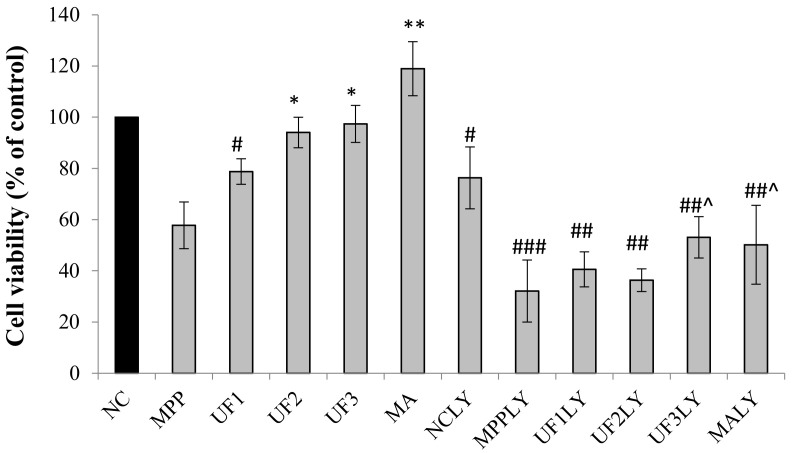
Effect of the samples on the neuronal cell viability induced by MPP^+^. ^#^ Vs NC *p* < 0.05; ^##^ Vs NC *p* < 0.01; ^###^ Vs NC *p* < 0.001; * Vs MPP *p* < 0.05; ** Vs MPP *p* < 0.01; *** Vs MPP *p* < 0.001; ^^^ Vs MPPLY *p* < 0.05; ^^^^ Vs MPPLY *p* < 0.01; ^^^^^ Vs MPPLY *p* < 0.001.

The results of protective effect of UF on MPP^+^-induced neurotoxicity in SH-SY5Y cells are shown in Figure 1. The exposure of the SH-SY5Y cells to 100-µM MPP^+^ significantly reduced cell viability to 60% compared with that of the normal cells. In contrast, pretreatment with different concentrations of UF reversed the decreased cell viability induced by MPP^+^. Co-incubation of UF at the dose of 500 µg/mL and 800 µg/mL increased the cell viability more than 30% compared with the MPP group. When exposed with PI3K inhibitor LY294002, the cell viability decreased in all groups compared with the NC group. The cell viability of NCLY and MPPLY group was nearly 80% and 32% compared with the NC group. Pretreatment with UF increased the cell viability to 55%—especially at the dose of 800 µg/mL.

**Figure 2:**
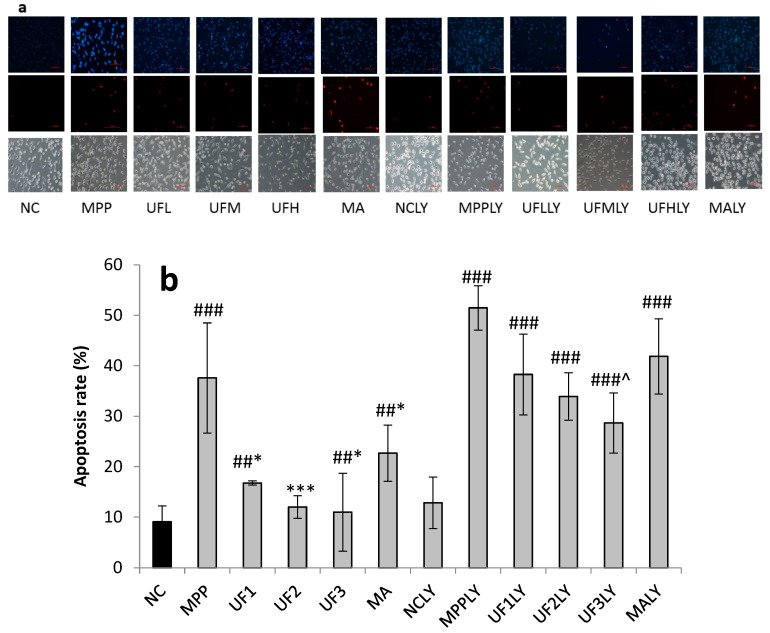

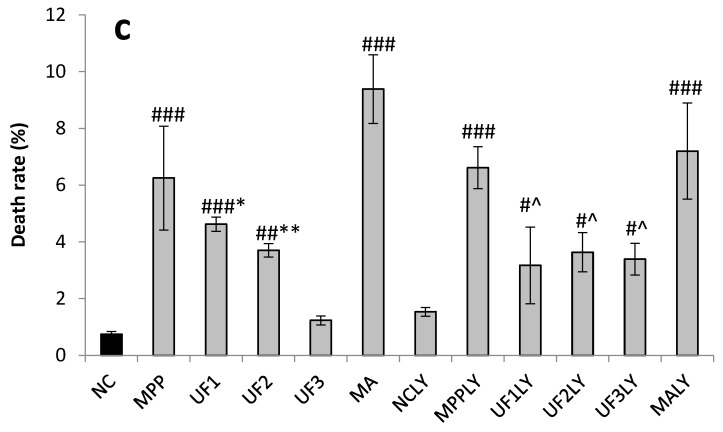
Nuclear morphology of MPP^+^ and UF treated SH-SY5Y cells for 48 h. (**a**) Protective effects of UF on MPP^+^-induced cell apoptosis rate %; (**b**) protective effects of UF on MPP^+^-induced cell Death rate % (**c**) Data are expressed as percentages and represent the mean ± SD of three separate experiments in which at least 200 cells were counted per one treatment group. ^#^ Vs NC *p* < 0.05; ^##^ Vs NC *p* < 0.01; ^###^ Vs NC *p* < 0.001; * Vs MPP *p* < 0.05; ** Vs MPP *p* < 0.01; *** Vs MPP *p* < 0.001; ^^^ Vs MPPLY *p* < 0.05; ^^^^ Vs MPPLY *p* < 0.01; ^^^^^ Vs MPPLY *p* < 0.001; Scale bar in the picture is 50 in length.

To determine whether the survival rate of SH-SY5Y neurons increased by UF treatment was related to cell apoptosis, cells were stained with the DNA dye Hoechst 33342/PI to visualize nuclear morphology (Figure 2). The results showed that incubation with MPP^+^ could cause SH-SY5Y cells apoptosis—including the degeneration of neuritis and shrinkage of cell bodies—as well as fragmentation and condensation of nuclei. Without exposure to MPP^+^, SH-SY5Y cells exhibited normal cellular morphology. Different doses of UF administration groups could reduce the apoptosis and death rate of SH-SY5Y cells (500 µg/mL and 800 µg/mL, respectively, *p* < 0.01), which indicated that the protective effect of UF on SH-SY5Y cells was related to reducing the apoptosis of SH-SY5Y cells. To determine whether the apoptosis of SH-SY5Y cells caused by MPP^+^ was related to the PI3K/AKT pathway, we added PI3K/AKT pathway inhibitor LY294002 during cell culture [7,8]. The degree of apoptosis and death rate of SH-SY5Y cells in MPPLY group increased significantly compared with NC group. Different concentrations of UF administration groups could reduce the apoptosis and death rate. The MPP^+-^induced apoptosis rate was 37.6%; the addition of LY294002 increased the frequency of apoptosis rate to 51.5%. UF at 800 µg/mL greatly reduced MPP^+^-induced apoptosis to 11.1%. With the addition of LY294002, the effect of UF at 800 µg/mL on MPP^+^-induced apoptosis was 28.7%. The apoptosis rate was different when cells were pretreatment with UF alone or together with LY294002 (11.1% versus 28.7%, *p* < 0.001). These data suggest that the protective effect of UF on SH-SY5Y cells was partly related to the PI3K/AKT pathway.

### 2.3. UF Effect on the Expression of PI3K, Akt and Its Phosphorylation

**Figure 3:**
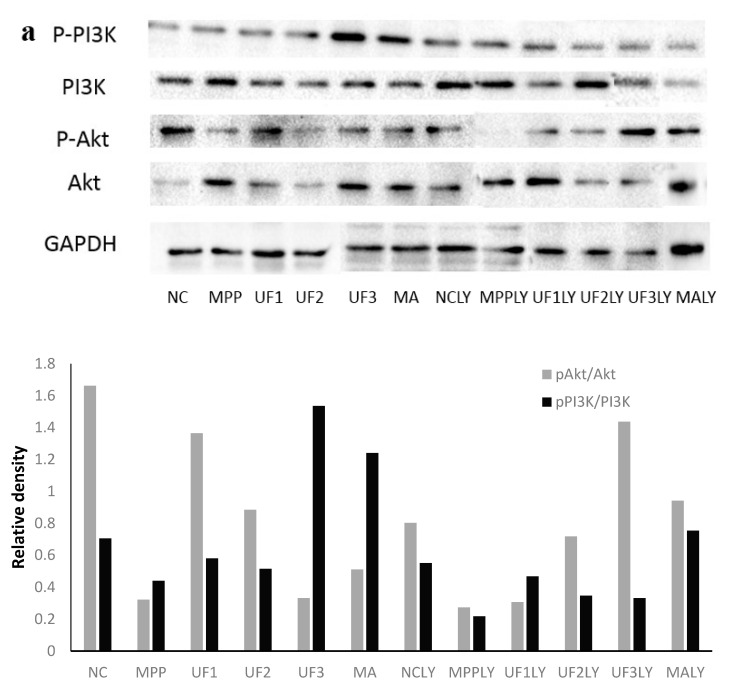

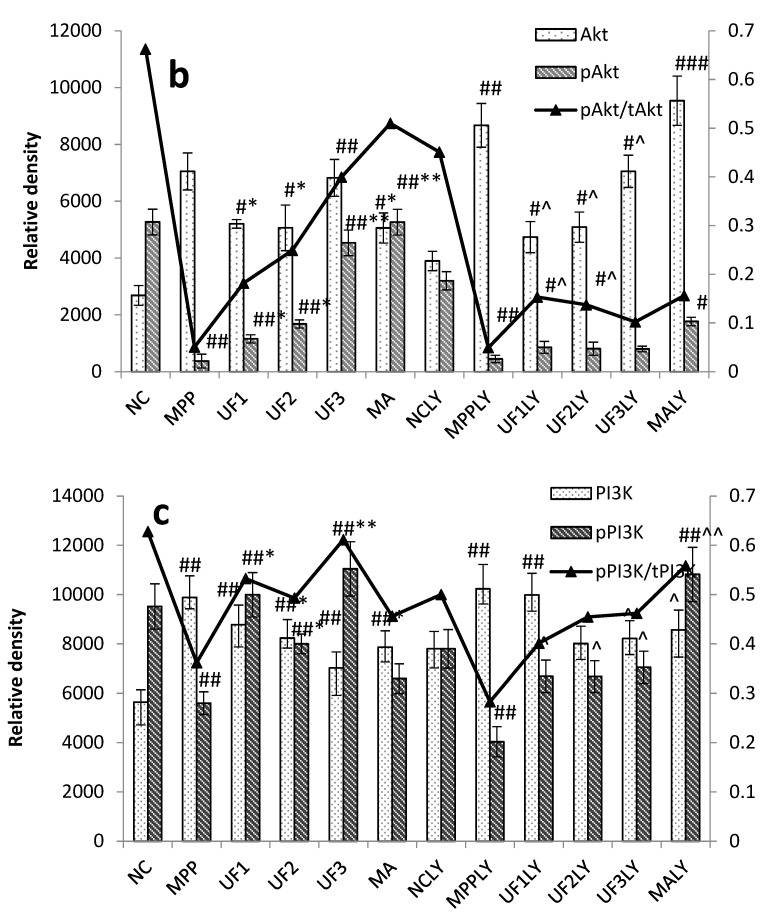
UF effect on the expression of PI3K, Akt and its phosphorylation. (**a**) PI3K, Akt and its phosphorylation results by western blot; (**b**) Akt and its phosphorylation results by immunochemistry method; (**c**) PI3K and its phosphorylation results by immunochemistry method; ^#^ Vs NC *p* < 0.05; ^##^ Vs NC *p* < 0.01; * Vs MPP *p* < 0.05; ** Vs MPP *p* < 0.01; ^^^ Vs MPPLY *p* < 0.05; ^^^^ Vs MPPLY *p* < 0.01.

Figure 3 summarizes the effect of the samples on the phosphorylation of PI3K and Akt proteins (). The immunochemistry results showed that MPP^+^ treatment decreased the phosphorylation of PI3K and Akt and inhibited the activation of PI3K/AKT pathway. Different concentrations of UF administration groups promoted the phosphorylation of PI3K and Akt, thereby activating the PI3K/AKT pathway (Figure 3b,c). The ratio of pAkt/tAkt and pPI3K/tPI3K were analyzed; the two ratios were lower in MPP group than in NC group. Different doses of UF treated increased the two ratios, respectively. We also examined whether the PI3K inhibitor LY294002 could inhibit the cytoprotective effect of UF. After incubation with LY294002, the degree of phosphorylated PI3K and Akt decreased significantly compared with NC group. Different concentrations of UF administration groups increased the phosphorylated PI3K and Akt level. Comparing the groups UF1 and UF1LY, UF2 and UF2LY, UF3 and UF3LY, the phosphorylated PI3K and Akt increased rates in UF1LY, UF2LY and UF3LY groups were lower than in the UF1, UF2 and UF3 groups, respectively. The results suggest that UF activated the PI3K/AKT pathway to inhibit the apoptosis of neuron cells and additional LY294002 alleviated UF neuron protective, but not completely. We tested the pAkt and pPI3K protein expression using western blotting to confirm. As we expected, results showed that the expression of pAkt and pPI3K was decreased in MPP group compared with the NC group. UF treatment groups reversed the MPP^+^ induced pAkt and pPI3K decrease markedly. After incubation with LY294002, the expression of pAkt and pPI3K were lower than the groups without treated with LY294002, respectively. Treatment with UF and positive drug MA increased the expression of pAkt and pPI3K at some extent. Therefore, it is plausible that the degradation pAkt and pPI3K may be regulated by UF and the UF treatment could activate PI3K–Akt pathway.

### 2.4. UF Effect on the Expression of NGF

**Figure 4:**
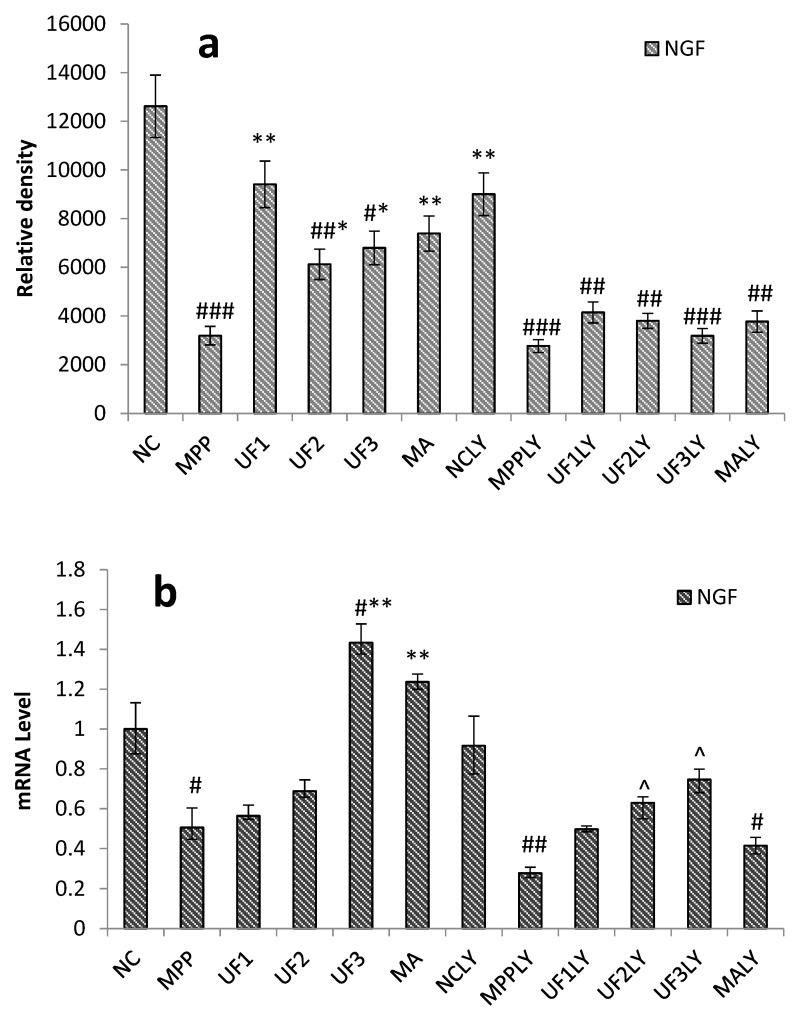
Protective effects of UF on MPP^+^-induced SH-SY5Y cell of NGF. (**a**) NGF results by immunochemistry method; (**b**) NGF results by RT-PCR method; ^#^ Vs NC *p* < 0.05; ^##^ Vs NC *p* < 0.01; ^###^ Vs NC *p* < 0.001; * Vs MPP *p* < 0.05; ** Vs MPP *p* < 0.01; ^^^ Vs MPPLY *p* < 0.05; ^^^^ Vs MPPLY *p* < 0.01.

To determine whether UF treatment could promote the survival of CNS neurons and activation PI3K/AKT pathway, we tested the effectiveness of UF on NGF. The protein expression level of the NGF decreased significantly in the MPP group, however, NGF expression elevated in different doses of the UF treatment groups, especially in the dose of 100-µg/mL UF group (Figure 4a). Moreover, the treatment of cells with 100-μM MPP^+^ and 20-μM LY294002 significantly decreased the mRNA level of NGF to 0.28 ± 0.03 of the NC group and the UF (800 µg/mL) combined with LY294002 treatment group increased that to 0.75 ± 0.06 of the NC group, respectively (Figure 4b).

### 2.5. UF Effect on the Expression of Apoptosis Related Proteins

**Figure 5:**
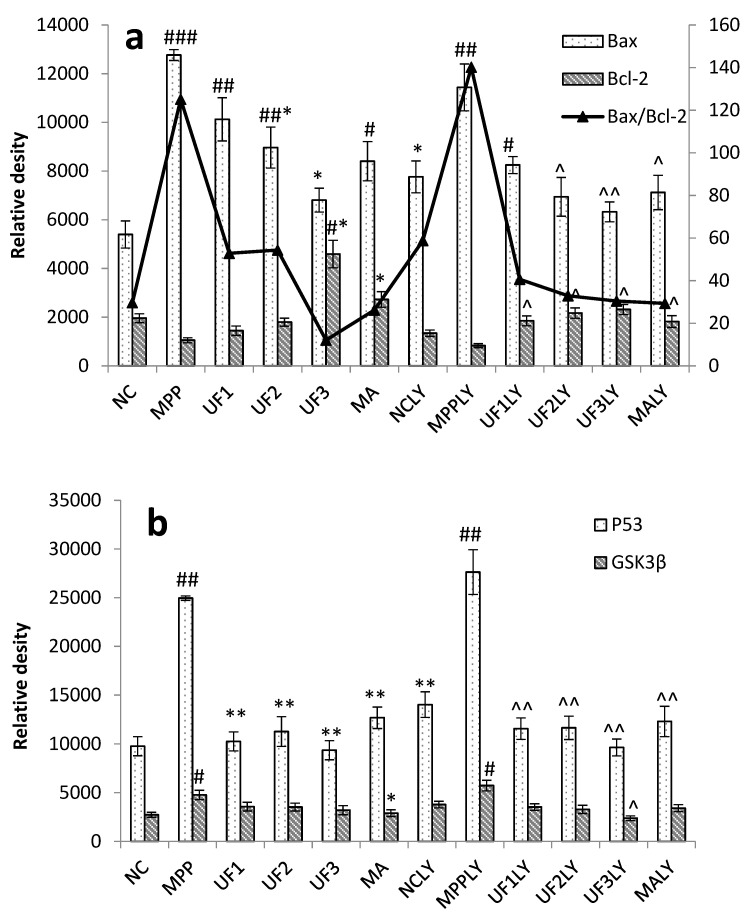

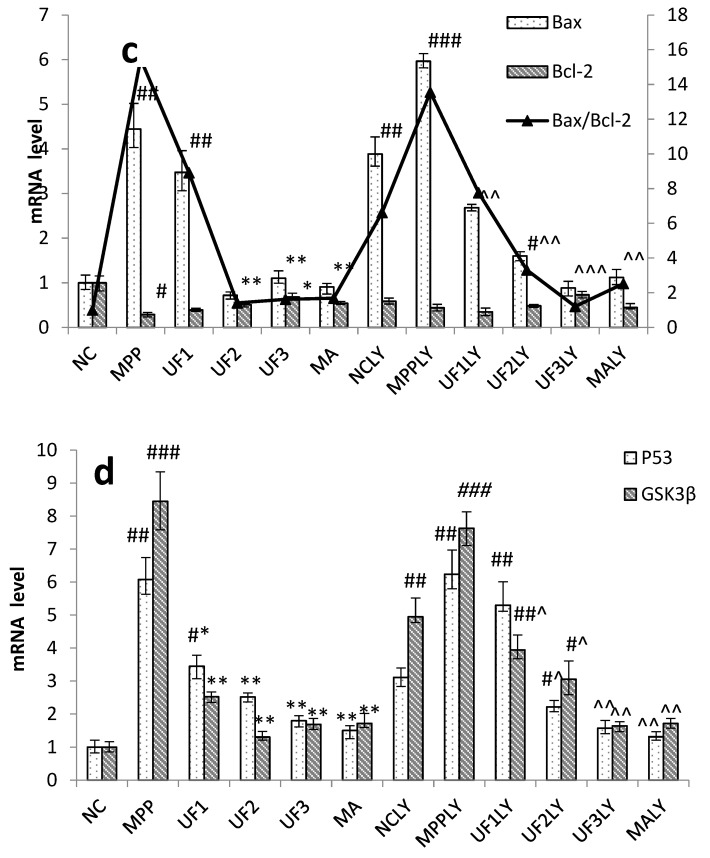
Protective effects of UF on MPP^+^-induced SH-SY5Y cell of apoptosis relative protein. (**a**) protein expression of Bcl-2 and Bax by immunochemistry method; (**b**) protein expression of p53 and GSK3β by immunochemistry method; (**c**) mRNA expression of Bcl-2 and Bax by RT-PCR method; (**d**) mRNA expression of Bcl-2 and Bax by RT-PCR method ^#^ Vs NC *p* < 0.05; ^##^ Vs NC *p* <0.01; ^###^ Vs NC *p* < 0.001; * Vs MPP *p* < 0.05; ** Vs MPP *p* < 0.01; ^^^ Vs MPPLY *p* < 0.05; ^^^^ Vs MPPLY *p* < 0.01; ^^^^^ Vs MPPLY *p* < 0.001.

Figure 5 summarizes apoptosis-related proteins in the PI3K/AKT pathway. From our data we concluded that UF has a certain inhibitory effect on the expression of pro-apoptotic proteins Bax, P53 and GSK3β, and also has a certain promotion effect on the anti-apoptotic protein Bcl-2 (Figure 5a,b). The ratio of Bax/Bcl-2 was increased to 4.2 and 4.7-fold in MPP and MPPLY groups compared with NC group, respectively. UF treatment group decreased the ratio of Bax/Bcl-2 at a dose dependent manner. The ratio of Bax/Bcl-2 in UF3 group was lower than in the NC group. The ratio of Bax/Bcl-2 in UF3LY group was nearly the same as in the NC group. The level of mRNA expression following MPP^+^ and UF treatment were examined by RT-PCR in order to confirm the protein changes in process of the cell apoptosis (Figure 5c,d). The PD model induced by MPP^+^ increased the mRNA expression level of pro-apoptosis protein such as Bax, p53 and GSK3β, and decreased the mRNA expression level of anti-apoptosis protein Bcl-2. However, there was a marked decrease in the mRNA expression level of pro-apoptosis protein levels and increase in the mRNA expression level of anti-apoptosis protein levels in the UF-treated groups. We also found that the mRNA expression of the key apoptosis-related proteins Bax, P53 and GSK3β was consistently upregulated to 5.97 ± 0.15, 6.24 ± 0.44 and 7.63 ± 0.52, respectively, in cells treated with 100-μM MPP^+^ and LY294002, whereas that of Bcl-2 was downregulated to 0.44 ± 0.07 of the control values. However, UF together with LY294002 treatment inhibited the up- or downregulation of Bax, P53, GSK3β and Bcl-2. Compared to the UF treated group, the up- or downregulation ability was weaker.

### 2.6. Caspase-3, -8 and -9 Activity

**Figure 6:**
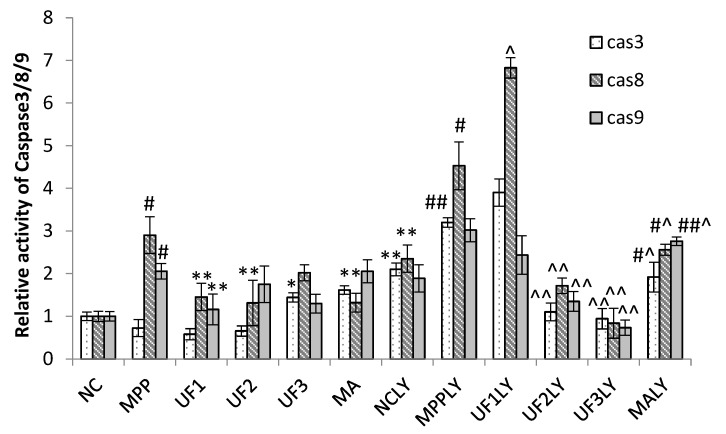
Protective effects of UF on MPP^+^-induced SH-SY5Y cell of relative activity of caspase-3, caspase-8 and caspase-9. ^#^ Vs NC *p* < 0.1; ^##^ Vs NC *p* < 0.01; * Vs MPP *p* < 0.1; ** Vs MPP *p* < 0.01; ^^^ Vs MPPLY *p* < 0.1; ^^^^ Vs MPPLY *p* < 0.01.

The treatment of cells with 100-μM MPP^+^ significantly increased the relative activity of caspase-3 (cas3), caspase-8 (cas8) and caspase-9(cas9) to 0.73 ± 0.2, 2.90 ± 0.43 and 2.06 ± 0.18 of the NC group, respectively (Figure 6); however, this significantly decreased to 0.58 ± 0.13, 1.45 ± 0.32 and 1.16 ± 0.36 of the NC group, respectively, under UF treatment at 100 μg/mL. Moreover, the treatment of cells with 100-μM MPP^+^ and 20-μM LY294002 significantly increased the relative activity of cas3, cas8 and cas9 to 3.21 ± 0.11 and 4.53 ± 0.56, 3.02 ± 0.27 of the NC group, respectively, which were much higher than MPP group; however, this significantly decreased to 0.94 ± 0.24, 0.84 ± 0.35 and 0.73 ± 0.18 of the control group, respectively, when treated with UF at 800-μg/mL and 20-μM LY294002. These results showed that UF treatment could promote the activity of caspase-3, caspase-8 and caspase-9 did not inhibited by LY294002.

## 3. Discussion

**Figure 7:**
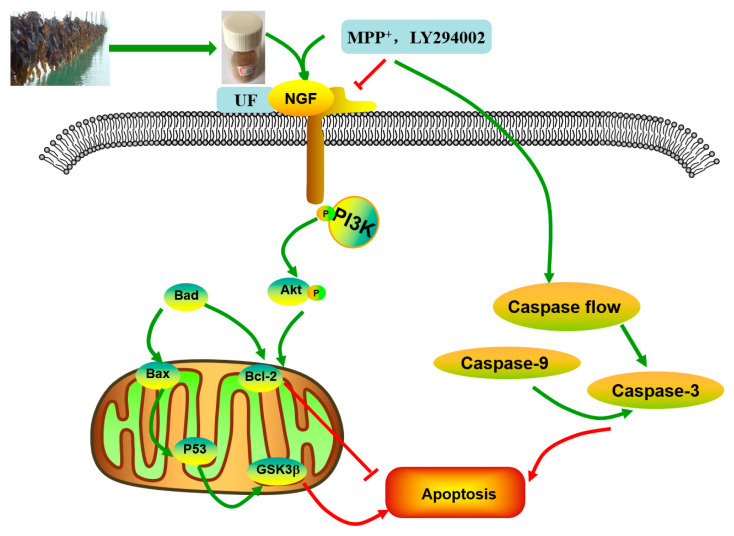
Effect of UF on MPP^+^-induced SH-SY5Y cells apoptosis.

PD is a neurodegenerative disease caused by both genetic and environmental factors. Neuroprotection therapeutic method caused interest in recently. Previous study found fucoidan extracted from brown seaweeds had neuron protective activity which related to its antioxidant activity [15]. Further studies found different fractions of fucoidan exhibited variety neuron protective activities. One fraction UF with higher uronic acid, lower sulfated group content and more complex monosaccharides exhibited the strongest activity. The neuron protective effect of UF may be mediated, in part, through antioxidant activity and the prevention of cell apoptosis [18]. The simplified depiction effect of UF on MPP^+^-induced cytotoxicity was summarized in Figure 7. UF treatment groups could decrease the cell apoptosis rate and increase the cell vitality. LY294002 could inactivate the PI3K–Akt pathway, thereby inhibiting cell proliferation and inducing apoptosis. The results found that the neuron protective effect of UF was alleviated when treated with LY294002. It is illustrated that the neuron protective effect of UF may relate with the PI3K–Akt pathway.

The PI3K–Akt pathway plays an important role in the survival and maintenance of many neuronal function such as long-term potentiation and memory formation. Inhibition of PI3K activity can offset the ability of nerve growth factors to promote cell survival [8]. Chondroitin sulfate (CS) in the cell matrix can protect SH-SY5Y cells by activating the PI3K–Akt signaling pathway [6]. Nerve growth factor (NGF) is a peptide molecule that plays a nutritional role in nerve cells. In the nervous system, NGF can increase the tolerance of cells under oxidative stress, which is the main mechanism involves NGF-induced activation of the PI3K–Akt pathway. Studies have shown that the application of corresponding treatments after spinal cord ischemia can induce NGF to activate the PI3K–Akt pathway to inhibit neuronal apoptosis [20]. Seow et al. found crude polysaccharides extracted from *Lignosus rhinocerotis* could stimulate neurogenesis without stimulating the production of NGF in PC-12 cells [21]. We found UF had a backbone of alternating 4-linked GlcA and 2-linked Man with the first Man residue from the nonreducing end accidentally sulfated at C6. UF and CS both have GluA and sulfate group, so we suppose UF could combine with NGF and increase the expression of the NGF, then activation the PI3K–Akt pathway. Our study confirmed that UF treatment could increase the expression of NGF, the effect was alleviated when added LY294002, but not disappeared completely. From our data, we suppose UF exhibited protective effect against MPP^+^-induced SH-SY5Y cell apoptosis by activating PI3K–Akt pathway through reacting with NGF. The chemical composition and structure of the polysaccharide had relationship with the effect on the NGF, chondroitin sulfate and fucoidan could increase the expression of the NGF protein, however, polysaccharide extracted from *Lignosus rhinocerotis* mimics the neurogenic activity of NGF.

PI3K is one of the signal molecules involved in intracellular signal transduction. When cells are stimulated by stimulating factors such as NGF, the phosphorylation of PI3K is activated. The present results demonstrated that UF treatment could enhance the phosphorylation of PI3K first, and then promoted the phosphorylation of Akt. This effect was alleviated when combined with LY294002.

When phosphorylating the PI3K and Akt, it activates or inhibits its downstream target proteins Bad, Bcl-2, Bax, caspase-9, GSK-3, mTOR, nuclear transcription factors, etc., in turn regulate cell proliferation, differentiation, apoptosis and invasion [22]. The influence of UF on these anti-apoptotic and pro-apoptotic protein were analyzed. Our study showed that UF could partially inhibit MPP^+^-induced dysfunction of the Bax/Bcl-2 system and decrease the expression of P53 and GSK3β protein, then inhibited cell apoptosis. This impact was alleviated when adding LY294002 in UF treated groups. As an anti-apoptotic member of the Bcl-2 family, Bcl-2 can bind Bax to form Bax/Bcl-2 heterodimers, thereby, attenuating the pro-apoptotic effect of Bax. Habaike *et al*. found polysaccharides extracted from *Laricifomes officinalis* Ames could attenuating cell apoptosis, increasing the ratio of Bcl-2/Bax and inhibiting cytochrome C release from mitochondria to cytosol in PC12 cells [23]. An acid-soluble polysaccharide (GFAP) prepared from *Grifola frondosa* could upregulate the expressions of Bax in HCC cells and induced the cell apoptosis [24]. The UF is a heteropolysaccharide with uronic acid and sulfate group, we suppose it can react with Bax and effect the formation of Bax/Bcl-2 heterodimers. P53 and GSK3β are thought to be a key factor in the subsequent apoptotic processes among the pro-apoptotic proteins. UF can react with these two proteins directly and reduce the cell apoptosis in the H_2_O_2_-reduced cell model [25].

Caspase family such as caspase-3 (cas3), caspase-8 (cas8) and caspase-9 (cas9) are crucial checkpoint in cell commitment to apoptosis. Cas3 is a critical executor of apoptosis being responsible for the proteolytic cleavage of many key proteins, which damage initiates the cell death program. Yu et al. found an acid-soluble polysaccharide could trigger apoptosis of HCC cells through mitochondria apoptotic pathway in a caspases-dependent pattern [24]. It means the acid polysaccharide could react with caspases-dependent pattern, it could decrease or increase the expression of caspase protein, which confirmed by our results. The addition of UF significantly attenuated the expression of cas3 in MPP^+^-induced SH-SY5Y cells, confirming UF had effect in terms of apoptosis process. LY294002 used did not lead to a complete inhibition of initiation-programmed cell death by UF treated groups, suggesting that other pathways are also involved. Cas8 and cas9 have shown increase in MPP^+^ and MPP^+^+LY294002 groups simultaneously, UF treated groups could decrease this rising tendency. The observed changes may suggest that UF could significantly decrease cas3, cas8 and casp9 activation, the consequence of which is apoptosis.

Our study showed that UF treatment could reversed the toxic effect of MPP^+^ on SH-SY5Y cells by activation the PI3K–Akt pathway. Addition of LY294002 significantly inhibited the PI3K–Akt pathway active and enhanced DNA fragmentation in SH-SY5Y cells, UF treated groups could still alleviate the cell apoptosis. In the present study, we demonstrated for the first time that UF attenuated the MPP^+^ induced apoptosis via reacting with NGF, Bax and cas3. Our data indicated that UF inhibited cell apoptosis by participating in PI3K–Akt pathway partially.

## 4. Materials and Methods

### 4.1. Preparation of Sulfated Polysaccharides

**Figure 8:**
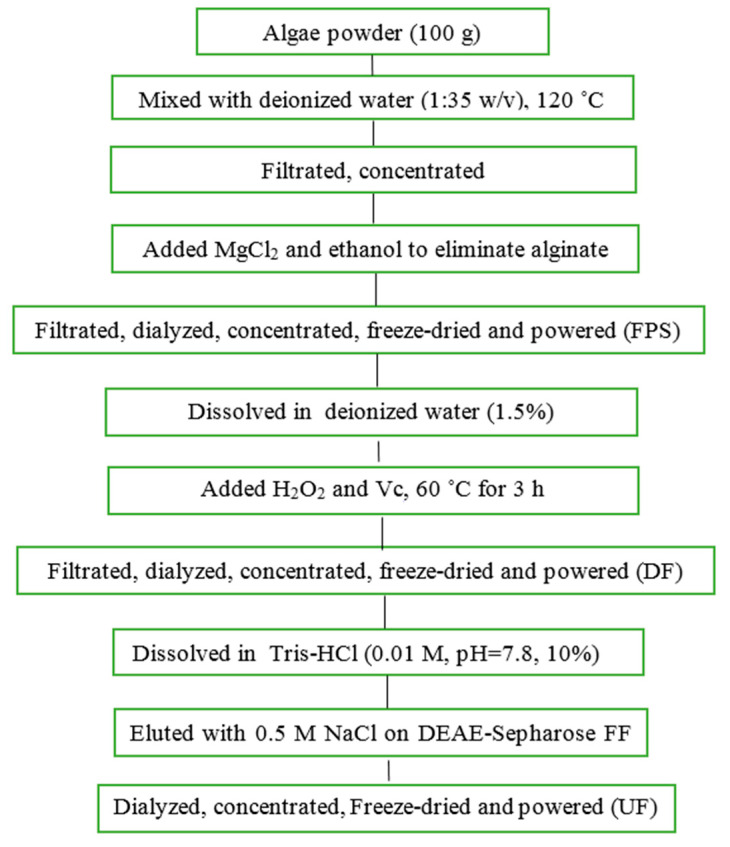
Flowchart of the extraction for UF.

*Saccharina japonica* (Laminariaceae), cultured along the coast of Dandong, China, was collected in August 2015, authenticated by Prof. Lanping Ding and stored as a voucher specimen (No. 83) in the Institute of Oceanology, CAS. The fresh algae were promptly washed, sun dried and kept in plastic bags at room temperature until use. FPS and UF were prepared according to our previous methods with minor modifications (Figure 8) [12,26]. Briefly, FPS was extracted in water solution at 120 ˚C using autoclave from *Saccharina japonica*. DF was prepared using ascorbate and hydrogen peroxide (30 mmol/L, 1:1) degradation method. UF were obtained using DEAE-Sepharose FF exchange chromatography previously described.

### 4.2. Analytical Methods

The total sugar content of samples were determined according to the method of Dubois et al. using L-fucose as the standard [27]. The sulfate content was analyzed by ion chromatography using K_2_SO_4_ as the standard. Uronic acid was estimated via a modified carbazole method using d-glucuronic acid as the standard [28]. The neutral sugar composition was determined by PMP-HPLC precolumn derivatization chromatography using ribose as interior label [29]. The molecular weight was assayed by an HP-GPC chromatography using a series of dextrans with different molecular weights as standards. A series of dextrans were purchased from the National Institute for the Control of Pharmaceutical and Biologic Products (China). Other standard reagents were purchased from Sigma-Aldrich (Milwaukee, WI, USA).

### 4.3. Cell Culture and Treatments

A dopaminergic cell line SH-SY5Y was used to establish an in vitro PD model. SH-SY5Ycells were kindly provided by Professor Ning Song (QingDao University) and maintained in Dulbecco’s modified Eagle medium/F12 supplemented with 10% newborn calf serum (Gibco) in an incubator with an atmosphere of 5% CO_2_ at 37 °C. For all experiments, the cells were seeded on 96-well plates, 24-well plates or 6-well plates at a density of 1 × 10^4^ cells–1 × 10^5^ cells/mL for 24 h. Then the cells were incubated with MPP^+^ for 30 min, then treated with different reagents for 24 h. The cells were divided into 12 groups. 1. NC group: treated with DMEM; 2: MPP group: treated with 100-µM MPP^+^; 3. UF1 group: treated with 100-µM MPP^+^ and 100-µg/mL UF; 4. UF2 group: treated with 100-µM MPP^+^ and 500-µg/mL UF; 5. UF3 group: treated with 100-µM MPP^+^ and 800-µg/mL UF; 6. MA group: treated with 100-µM MPP^+^ and 100-mM positive drugs Modopar; 7. NCLY group: treated with DMEM and 20-µM LY294002; 8: MPPLY group: treated with 100-µM MPP^+^ and 20-µM LY294002; 9. UF1LY group: treated with 100-µM MPP^+^, 100-µg/mL UF and 20-µM LY294002; 10. UF2LY group: treated with 100-µM MPP^+^, 500-µg/mL UF and 20-µM LY294002; 11. UF3LY group: treated with 100-µM MPP^+^, 800-µg/mL UF and 20-µM LY294002; 12. MALY group: treated with 100-µM MPP^+^, 100-mM positive drugs Modopar and 20-µM LY294002. All the group had six wells and all the experiments were repeated three times in different batches of cells.

### 4.4. Measurement of Cell Viability by MTT

SH-SY5Y cells were plated at a density of 1 × 10^4^ cells/100 µL in 96-well plates. Cell viability was quantitatively assessed using the MTT ([3-(4,5-dimethyl-2-thiazolyl)-2,5-diphenyl tetrazolium bromide]) assay [9]. After 24 h treatment, 20 µL MTT (0.5 mg/mL) regent was added to each well and incubated at 37 °C for 4 h. The medium was removed and washed twice with phosphate buffer solution (pH 7.4), then 200 µL DMSO was added to solubilize the formazan crystals. Cell viability was measured at 494 nm by spectrophotometer (Bio-Tec Gen 5, Winooski, VT, USA). Unless stated otherwise, all other chemicals were purchased from Sigma-Aldrich.

### 4.5. Observation of Morphologic Changes

Cells were seeded in 24-well plates at a density of 1 × 10^5^ for 24 h. After treatment for 24 h, cells were washed with phosphate-buffered saline and stained with 400 µL Hoechst 33,342 (2.5 µg/mL) for 5 min in the dark. After removing the medium and washed twice with phosphate buffer solution (pH 7.4), 400 µL PI (12.5 µg/mL) was added for 5 min in the dark. Cells with typical apoptotic nuclear morphology such as nuclear shrinkage and fragmentation and micronuclei formation were identified under fluorescent microscope and counted using randomly selected fields on numbered slides. The percentage of apoptotic cells was scored by counting at least 200 cells per treatment group and the average percentage of apoptotic cells was determined for each UF treatment and expressed as the mean ± SD.

### 4.6. Immunocytochemistry

Immunocytochemistry was performed and modified according to Iida’s study. Briefly, SH-SY5Y cells were seeded in 12-wells plates and incubation with MPP^+^ and different reagent for 24 h. After washing with PBS for three times and fixing with PBS containing 4% (wt/vol) paraformaldehyde for 15 min and then permeabilized with 0.5% (wt/vol) Triton X-100 and blocked with 5% goat serum for 1 h at room temperature. Subsequently, the cells were incubated with rabbit monoclonal anti-Akt (1:200), anti-PI3K (1:200), anti-Bax (1:100), anti-Bcl-2 (1:100), anti-GSK3β (1:200), anti-pPI3K (1:200), anti-p53 (1:100), anti-NGF (1:200), anti-pAkt (1:200) antibodies at 4 °C overnight. After washing with PBST and incubated with the second antibody (1:200) in PBST for 1 h. After the samples were washed with PBS three times, they were embedded in DAPI for 5 min and then washed with PBST 4 times. The images were obtained using an Olyba microscope. The mean fluorescence intensity was calculated by Image-Pro (Rockville, MD, USA).

### 4.7. Western Blot Analysis

Cells were lysed in lysis solution (Ambion, Grand Island, NY, USA) and incubated at 95 °C for 10 min. Protein concentration was determined by the Bradford assay kit (Takara Biotechnology, Dalian, China). Twenty micrograms of total proteins was separated by 10%–12% sodium dodecyl sulfate polyacrylamide gels and then transferred to polyvinylidene difluoride membranes. Blots were probed with rabbit monoclonal anti-Akt (1:1000), anti-pAkt (1:1000), anti-PI3K (1:1000), anti-pPI3K (1:1000). Blots were also probed with rabbit monoclonal anti-GAPDH antibody (Milwaukee, WI, USA, Sigma, 1:10,000) as a loading control. Anti-rabbit secondary antibodies conjugated to horseradish peroxidase were used at 1:10,000 (Santa Cruz Biotechnology, Santa Cruz, CA, USA). UVP BioSpectrum^®^CCD imaging system (Davis, CA, USA) was used for imaging and analysis. Camera settings were manipulated in preview mode to optimize the exposure and determine the appropriate final exposure settings. Exposures of 30 s up to 5 min were used for data collection. Results were analyzed through scanning densitometry by UVP Vision Works LS Software (UVP, Cambridge, UK).

### 4.8. Total RNA Extraction and Real Time PCR

SH-SY5Y Cells were seeded in 6-wells plates and incubation with MPP^+^ and different reagent for 24 h. Total RNA was isolated by Trizol Reagent (Takara Biotechnology, Dalian, China) ccording to the manufacturer’s instructions. From each sample, 1 µg of total RNA was retrotranscripted into cDNA (Takara RR047A, Dalian, China). Then, 2 µL of each sample was used as a template for amplification reactions conducted with the SYBR Premix Ex TaqTMⅡ (Takara Biotechnology, Dalian, China) following the manufacturer’s instructions. The PCR amplifications were conducted using a life Technology 7500 fast Real-time PCR system. The expression of house-keeping gene, GAPDH mRNA, was served as the standardized control. Primer (showed in Table 2) selection was performed using the Primer Premier Design Software, version 1.0 (Idaho Technology, Inc., Alameda, CA, USA). The mRNA level for the control group was set as 100%.

### 4.9. Caspase-3, -8 and -9 Activity

After treatment of cells with UF for 24 h, the cells were harvested using cell scrapers and washed in ice-cold PBS. Then, the cells were lysed for 30 min on the ice in 100 µL of Cell Lysis Reagent supplemented with complete protease inhibitor cocktail. The protein concentration of cell lysates was determined by Bicinchoninic acid (BCA) assay (Takara Biotechnology, Dalian, China).

### 4.10. Statistical Analysis of Data

The data are presented as the mean values ± 1 SD (*n* = 8−10). The data were analyzed by a one-way ANOVA, a Duncan’s multiple-range test and an LSD test at a significance level of *p* < 0.05. SPSS 22.0 software (New York, NY, USA) was used for the analysis.

## 5. Conclusions

Fucoidan is a kind of sulfated polysaccharide extracted from brown algae. UF is a heteropolysaccharide purified from fucoidan. In our previous study, we found UF had excellent neuron protective activity. However, the mechanism is still unknown. In our present study, we demonstrated that UF could act on the extracellular growth factor NGF and cause progressive activation of the PI3K–Akt pathway. Furthermore, this study provides further insight into the mechanisms of UF, including regulating downstream signaling molecules and proteins of the PI3K–Akt pathway and the a alleviate effect by adding LY294002, which means the neuron protective activity of UF was partly through PI3K–Akt pathway. These findings contribute to a better understanding of the critical roles of UF in treating PD and may elucidate the molecular mechanisms of UF effects in PD.

## Figures and Tables

**Table 1:** The yield (% compared with the dry *S. japonica*), chemical composition (% dry weight) and molecular weight of fucoidan (FPS), DF and its fraction (UF) isolated from *S. japonica.*

Sample	Yield%	Fucose	Uronic Acid	Sulfate	Molecular Weight (Da)	Neutral Sugar (Mole Ratio) ^a^					
						Fuc	Gal	Man	Glc	Rha	Xyl
FPS	2.2	26.12	4.93	28.01	174,000	1.00	0.39	0.098	0.031	0.091	0.022
DF	1.3	28.7	3.65	30.1	9500	1.00	0.58	0.038	0.16	0.054	0.033
UF	0.3	19.12	14.25	21.21	6544	1.00	0.71	0.11	0.26	0.11	0.087

^a^ Fuc: fucose; Gal: galactose; Man: mannose; Glc: glucose; Rha: rhamnose; Xyl: xylose.

**Table 2:** Primers used for real-time RT-PCR.

Target Gene		Primer Sequences	Amplicon (bp)
Bax	FP	GGCGAATTGGACATGAAC	182
	RP	CCGAAGTAGGAGAGGAGG	
Bcl-2	FP	CCCCAGAAGAAACTGAACC	195
	RP	GCATCTCCTTGTCTACGC	
GSK3β	FP	ATTCCCTCAAATTAAGGCACCTCC	142
	RP	ATACTCCAGCAGACGGCTACACAG	
p53	FP	GGCGAATTGGAGATGAAC	156
	RP	CCGAAGTAGGAGAGGAGG	
GAPDH	FP	TTCACCACCATGGAGAAGGC	247
	RP	GGCATGGACTGTGGTCATGA	
NGF	FP	TCCAGGTGCATAGCGTAATG	195
	RP	CTCCGGTGAGTCCTGTTGAA	

## References

[1] Mariani S., Ventriglia M., Simonelli I., Bucossi S., Siotto M., Donno S., Vernieri F., Squitti R. (2016). Association between sex, systemic iron variation and probability of Parkinson’s disease. Int. J. Neurosci..

[2] Sarrafchi A., Bahmani M., Shirzad H., Rafieian-Kopaei M. (2015). Oxidative stress and Parkinson’s disease: New hopes in treatment with herbal antioxidants. Curr. Pharm. Des..

[3] Naughton C., Moriarty N., Feehan J., O’Toole D., Dowd E. (2016). Differential pattern of motor impairments in neurotoxic, environmental and inflammation-driven rat models of Parkinson’s disease. Behav. Brain Res..

[4] Tatton N.A., Maclean-Fraser A., Tatton W.G., Perl D.P., Olanow C.W. (1998). A fluorescent double-labeling method to detect and confirm apoptotic nuclei in Parkinson’s disease. Ann. Neurol..

[5] Tatton W.G., Chalmers-Redman R., Brown D., Tatton N. (2003). Apoptosis in Parkinson’s disease: Signals for neuronal degradation. Ann. Neurol..

[6] Perier C., Bove J., Vila M. (2012). Mitochondria and Programmed Cell Death in Parkinson’s Disease: Apoptosis and Beyond. Antioxid. Redox Signal..

[7] Cheung E.C.C., Melanson-Drapeau L., Cregan S.P., Vanderluit J.L., Ferguson K.L., McIntosh W.C., Park D.S., Bennett S.A.L., Slack R.S. (2005). Apoptosis-inducing factor is a key factor in neuronal cell death propagated by BAX-dependent and BAX-independent mechanisms. J. Neurosci..

[8] Yao R., Cooper G.M. (1995). Requirement for phosphatidylinositol-3 kinase in the prevention of apoptosis by nerve growth factor. Science.

[9] Abarikwu S.O., Farombi E.O., Pant A.B. (2011). Biflavanone-kolaviron protects human dopaminergic SH-SY5Y cells against atrazine induced toxic insult. Toxicol. In Vitro.

[10] Ale M.T., Mikkelsen J.D., Meyer A.S. (2011). Important Determinants for Fucoidan Bioactivity: A Critical Review of Structure-Function Relations and Extraction Methods for Fucose-Containing Sulfated Polysaccharides from Brown Seaweeds. Mar. Drugs.

[11] Wijesinghe W.A., Jeon Y.-J. (2012). Biological activities and potential industrial applications of fucose rich sulfated polysaccharides and fucoidans isolated from brown seaweeds: A review. Carbohydr. Polym..

[12] Wang J., Zhang Q., Zhang Z., Li Z. (2008). Antioxidant activityof sulfated polysaccharide fractions extracted from Laminaria japonica. Int. Biol. Macromol..

[13] Liu H.D., Wang J., Zhang Q.B., Zhang H. (2018). The effect of different substitute groups and molecular weights of fucoidan on neuroprotective and anticomplement activity. Int. J. Biol. Macromol..

[14] Cui Y.Q., Zhang L.J., Zhang T., Luo D.Z., Jia Y.J., Guo Z.X., Zhang Q.B., Wang X., Wang X.M. (2010). Inhibitory effect of fucoidan on nitric oxide production in lipopolysaccharide-activated primary microglia. Clin. Exp. Pharmacol. Physiol..

[15] Luo D., Zhang Q., Wang H., Cui Y., Sun Z., Yang J., Zheng Y., Jia J., Yu F., Wang X. (2009). Fucoidan protects against dopaminergic neuron death in vivo and in vitro. Eur. J. Pharmacol..

[16] Cui Y.Q., Jia Y.J., Zhang T., Zhang Q.B., Wang X.M. (2012). Fucoidan Protects against Lipopolysaccharide-Induced Rat Neuronal Damage and Inhibits the Production of Proinflammatory Mediators in Primary Microglia. Cns Neurosci. Ther..

[17] Jin W., Wang J., Jiang H., Song N., Zhang W., Zhang Q. (2013). The neuroprotective activities of heteropolysaccharides extracted from Saccharina japonica. Carbohydr. Polym..

[18] Wang J., Liu H., Jin W., Zhang H., Zhang Q. (2016). Structure-activity relationship of sulfated hetero/galactofucan polysaccharides on dopaminergic neuron. Int. J. Biol. Macromol..

[19] Jin W., Wang J., Ren S., Song N., Zhang Q. (2012). Structural Analysis of a Heteropolysaccharide from Saccharina japonica by Electrospray Mass Spectrometry in Tandem with Collision-Induced Dissociation Tandem Mass Spectrometry (ESI-CID-MS/MS). Mar. Drugs.

[20] Patapoutian A., Reichardt L.F. (2001). Trk receptors: Mediators of neurotrophin action. Curr. Opin. Neurobiol..

[21] Seow S.L.S., Eik L.F., Naidu M., David P., Wong K.H., Sabaratnam V. (2015). Lignosus rhinocerotis (Cooke) Ryvarden mimics the neuritogenic activity of nerve growth factor via MEK/ERK1/2 signaling pathway in PC-12 cells. Sci. Rep..

[22] Manning B.D., Cantley L.C. (2007). AKT/PKB Signaling: Navigating Downstream. Cell.

[23] Habaike A., Yakufu M., Cong Y., Gahafu Y., Li Z., Abulizi P. (2020). Neuroprotective effects of Fomes officinalis Ames polysaccharides on A beta(25-35)-induced cytotoxicity in PC12 cells through suppression of mitochondria-mediated apoptotic pathway. Cytotechnology.

[24] Yu J., Liu C., Ji H.-Y., Liu A.-J. (2020). The caspases-dependent apoptosis of hepatoma cells induced by an acid-soluble polysaccharide from Grifola frondosa. Int. J. Biol. Macromol..

[25] Wang J., Liu H.D., Zhang X., Li X.P., Geng L.H., Zhang H., Zhang Q.B. (2017). Sulfated Hetero-Polysaccharides Protect SH-SY5Y Cells from H2O2-Induced Apoptosis by Affecting the PI3K/Akt Signaling Pathway. Mar. Drugs.

[26] Wang J., Wang F., Zhang Q., Zhang Z., Shi X., Li P. (2009). Synthesized different derivatives of low molecular fucoidan extracted from Laminaria japonica and their potential antioxidant activity in vitro. Int. J. Biol. Macromol..

[27] Dubois M., Gilles K.A., Hamilton J.K., Rebers P.T., Smith F. (1956). Colorimetric Method for Determination of Sugars and Related Substabces. Anal. Chem..

[28] Bitter T., Muir H.M. (1962). A modified uronic acid carbazole reaction. Anal. Biochem..

[29] Honda S., Akao E., Suzuki S., Okuda M., Kakehi K., Nakamura J. (1989). High-performance liquid chromatography of reducing carbohydrates as strongly ultraviolet-absorbing and electrochemically sensitive 1-phenyl-3-methyl5-pyrazolone derivatives. Anal. Biochem..

